# Role of generative artificial intelligence in publishing. What is acceptable, what is not.

**DOI:** 10.1051/ject/2023033

**Published:** 2023-09-08

**Authors:** Raymond Wong

Without a doubt, Generative Artificial Intelligence (AI) is a hot topic in the media in recent times. This was spurred initially by the popular, widespread use of ChatGPT and other platforms to generate written material, imagery, and even audio/visual productions based on user-inputted prompts. Generative AI is defined by AI as: “a type of AI that uses machine learning algorithms to create new and original content like images, video, text, and audio” [1].

How do these technological advancements impact us in the scientific publishing world? Specifically, when is it appropriate and perhaps more importantly, when is it NOT appropriate to use such tools in producing published scientific articles?

Strictly speaking, every time a word processor suggests a better way to phrase a sentence, basic AI is being applied in one’s writing. Taken to a much more sophisticated level, a writer can submit a roughly written draft to a generative AI platform using large language models (LLMs) and a more sophisticated written output could be produced and ultimately submitted. If a student in an English class, meant to teach students how to write well did submit such a piece for an assignment, this use of AI might constitute cheating. However, when authors use AIs to help polish their work for publication, this should be entirely appropriate since such an application enhances the work to help readers comprehend and appreciate such work better. Our journal has recently started providing our authors the option of using a “comprehensive writing and publication assistant” to improve their submissions. Submitting authors should see a link to the service we are partnering with the Paperpal Preflight Screening Tool. For a very reasonable fee, the tool offers translation, paraphrasing, consistency, and journal submission readiness checks on uploaded manuscript drafts. This service is particularly helpful for some international authors who may have a challenging time meeting our language requirement standards.

In another scenario applicable to publishing, say a peer reviewer wishes to use AI to evaluate a submission. You might be asking: “wait, can AI do that?” Most certainly! Would that be acceptable though? There are indeed platforms out there that are trained on publicly available biomedical publications such that the AI is able to look up references to help a peer reviewer assess manuscripts. Maybe the peer reviewer just needs help getting started with the first draft of their review, or they may feel that the author’s language skills need a lot of help like in the earlier scenario. A major difference here, however, is that when a peer reviewer uploads a manuscript on one of these platforms, they would be breaching confidentiality which is not acceptable. The NIH does not allow AI to be used for the peer review of grant applications [2], and neither should such technology be used for publication peer reviews because the same breach of confidentiality occurs when an author’s manuscript is uploaded to a third party’s platform. Other concerns that Hosseini and Horbach (2023) identified fault “the fundamental opacity of LLM’s training data, inner workings, data handling, and development processes” which can result in “potential biases and the reproducibility of review reports” [3]. JECT peer reviewers will therefore be instructed to not rely on such systems in conducting their evaluations. Moreover, no editorial decisions on the final outcome of any manuscripts will be made using AI tools alone.

To help authors navigate this new terrain, JECT will endeavor to provide new guidance in our Instruction for Authors as other journals are currently implementing [4]. Some principles that other journals are recommending and that we will likely adopt include:

1. Authors should provide a declaration of any AI-generated content in the submission and include the details of the tool or system used such as the Paperpal example above. These declarative statements should be made whether generative AI technology is used in the writing process or to generate tables, figures, and even videos. Basic tools for checking grammar, spelling and references are exempted.
2. Authors confirm that they have reviewed the product generated by the AI systems and are ultimately responsible and accountable for the integrity of such content.
3. AI systems cannot be attributed as authors. Only humans can.

As many other journals do, JECT uses other AI-like tools, such as for similarity checks but will always apply human review and oversight. In the future, other tools might become available for journals to use in ways that improve quality and efficiency. Being in the early stages of this new era, generative AI technology and its uses will continue evolving, raising new issues and concerns that have yet to be encountered or addressed. The Committee on Publication Ethics (COPE) has provided guidelines for the use of AI tools by authors, peer reviewers, editors, and publishers in scholarly publications; and will probably continue to contribute by providing guidance in the future [5]. Ultimately, full transparency, responsibility, and accountability are the appropriate primary principles to be adhered to as we continue in our mission to disseminate trusted information within our sphere of perfusion sciences.
